# Research on Blood Cell Image Detection Method Based on Fourier Ptychographic Microscopy

**DOI:** 10.3390/s25030882

**Published:** 2025-01-31

**Authors:** Mingjing Li, Le Yang, Shu Fang, Xinyang Liu, Haijiao Yun, Xiaoli Wang, Qingyu Du, Ziqing Han, Junshuai Wang

**Affiliations:** College of Electronic Information Engineering, Changchun University, Changchun 130022, China; limj@ccu.edu.cn (M.L.); 230402191@mails.ccu.edu.cn (L.Y.); 230402186@mails.ccu.edu.cn (X.L.); yunhj@ccu.edu.cn (H.Y.); wangxl@ccu.edu.cn (X.W.); 230401175@mails.ccu.edu.cn (Q.D.); 230401178@mails.ccu.edu.cn (Z.H.); 240401173@mails.ccu.edu.cn (J.W.)

**Keywords:** Fourier ptychographic microscopic imaging, YOLOv7 (You Only Look Once version 7), blood cell detection, feature fusion

## Abstract

Autonomous Fourier Ptychographic Microscopy (FPM) is a technology widely used in the field of pathology. It is compatible with high resolution and large field-of-view imaging and can observe more image details. Red blood cells play an indispensable role in assessing the oxygen-carrying capacity of the human body and in screening for clinical diagnosis and treatment needs. In this paper, the blood cell data set is constructed based on the FPM system experimental platform. Before training, four enhancement strategies are adopted for the blood cell image data to improve the generalization and robustness of the model. A blood cell detection algorithm based on SCD-YOLOv7 is proposed. Firstly, the C-MP (Convolutional Max Pooling) module and DELAN (Deep Efficient Learning Automotive Network) module are used in the feature extraction network to optimize the feature extraction process and improve the extraction ability of overlapping cell features by considering the characteristics of channels and spatial dimensions. Secondly, through the Sim-Head detection head, the global information of the deep feature map (mean average precision) and the local details of the shallow feature map are fully utilized to improve the performance of the algorithm for small target detection. MAP is a comprehensive indicator for evaluating the performance of object detection algorithms, which measures the accuracy and robustness of a model by calculating the average precision (AP) under different categories or thresholds. Finally, the Focal-EIoU (Focal Extended Intersection over Union) loss function is introduced, which not only improves the convergence speed of the model but also significantly improves the accuracy of blood cell detection. Through quantitative and qualitative analysis of ablation experiments and comparative experimental results, the detection accuracy of the SCD-YOLOv7 algorithm on the blood cell data set reached 92.4%, increased by 7.2%, and the calculation amount was reduced by 14.6 G.

## 1. Introduction

As science and technology continue to evolve, medical imaging technology has become a crucial component in both clinical diagnosis and biomedical research [1]. This technology is provided to physicians as a robust tool for studying, monitoring, and diagnosing diseases [2]. By utilizing these technologies, medical professionals can gain a more profound understanding of the pathological process, thereby enabling them to provide patients with more precise treatment plans. Clinical studies have demonstrated that when the human body is afflicted with diseases, there are corresponding changes in the number and structure of blood cells [3]. A significant basis for disease diagnosis can be served by these alterations.

Microscopic imaging detection is a prevalent method in pathology, which is crucial for examining the microstructure and cell morphology of biological tissues [4]. The performance of conventional microscopes is constrained by the diffraction effect of light. To achieve higher resolution, it is essential to increase the numerical aperture (NA) of the microscope objective. However, this approach reduces the imaging field of view, which is detrimental for studies requiring an overview of the entire smear.

In order to address this issue, Fourier Stacked Microcomputed Tomography (FPM) is employed in this paper to gather images and construct datasets. Subsequently, a deep neural network is utilized as the training model [5]. An enhanced algorithm for blood cell detection is proposed in the paper. The distinctive imaging capabilities of FPM have significantly advanced biomedical research, enhancing the efficiency of blood cell detection and providing a more comprehensive diagnostic basis for physicians. Through deep learning, blood cell images can be automatically analyzed and identified, assisting doctors in making preliminary assessments and early predictions of patients’ conditions [6]. This has significant medical diagnostic implications.

An introduction to the overarching research background, rationale, and objectives is provided in Section 1 of this paper. A thorough examination of select works relevant to the article is presented in Section 2. The methodology and architectural framework proposed are detailed in Section 3. The experimental process is elucidated in Section 4, and the results obtained are discussed. The conclusion is succinctly summarized in Section 5.

## 2. Related Works

### 2.1. Fourier Microscopic Imaging

The optical microscope is a traditional tool for microstructure analysis, consisting of core components such as the camera, lens, and lens barrel [7]. The performance of this instrument is typically gauged by two key indicators: resolution and field of view. Resolution is primarily influenced by the numerical aperture of the lens, while the field of view is determined by the aperture size of each lens within the system [8]. In microscopic imaging systems, it is often challenging to optimize both spatial resolution and field of view simultaneously due to an inherent trade-off between them. To capture the minute characteristics of a sample, it is necessary to increase the numerical aperture of the lens to enhance resolution [9]. However, this results in a narrowing of the field of view. Using a low-magnification lens allows for the acquisition of a comprehensive view of the sample, but details are difficult to discern. Conversely, a high-magnification lens can reveal detailed features of the sample, but at the expense of observing the overall structure.

In 2013, Professor Zheng Guoan and his team at the California Institute of Technology pioneered a groundbreaking microscopic imaging technology known as FPM [10]. This technology offers an expanded field of view and superior resolution, surpassing the constraints of conventional optical microscopes [11]. By leveraging synthetic aperture technology and optimization theory, high-quality, high-resolution images can be achieved even with low-magnification lenses. Since its inception, FPM has been swiftly adopted across various domains, including biological sample imaging, cell detection, counting, and digital pathology. In recent years, significant advancements have been made in FPM’s implementation methods, imaging performance, and reconstruction efficiency. These developments not only underscore the immense potential of FPM in the biomedical realm but also highlight its promise for sustainable growth [12,13].

Compared with the images obtained by using a standard optical microscope, confocal microscope, and standard dye, FPM technology has the advantages of high resolution and large field of view, no need for dyeing or low dyeing demand, high acquisition efficiency, and wide applicability.

The advent of FPM technology offers a robust solution to the challenges inherent in the current field of blood cell image acquisition [14]. By subtly modifying the structure of the light source, this technology not only markedly improves the clarity of conventional microscope images but also streamlines the experimental procedure. The design of the FPM light source is both straightforward and cost-efficient, capable of producing high-resolution white blood cell images with an expansive field of view [15,16]. Leveraging the benefits of this technology in conjunction with the superior performance of deep learning networks in target recognition tasks, we have successfully achieved precise detection of blood cells within the bloodstream [17].

### 2.2. Blood Cell Detection

The conventional method of microscope detection not only demands substantial human and material resources but also leaves room for subjective bias in the results [18]. While current blood cell analysis instruments offer rudimentary cell count data, they fall short in examining the morphological attributes of blood cells, thereby constraining their utility in adjunctive medical diagnoses [19]. However, with the swift advancements in digital and deep learning technologies within computer vision, the integration of computer graphics has furnished physicians with a novel tool to ascertain both the number and morphology of blood cells with heightened precision [20]. This represents a significant milestone in this domain.

The international exploration of red blood cell counting methods commenced in 1852 [21]. Three years later, in 1855, a specialized counting plate for blood cell analysis was invented. Currently, two primary technical approaches are employed for blood cell detection: one utilizes image processing technology, while the other employs deep learning techniques for analysis [22].

#### 2.2.1. Methods Based on Image Processing

Cuevas et al. developed an algorithm for the automatic recognition of white blood cells in complex images, optimizing the coding of candidate ellipses using a DE algorithm to adapt to the white blood cells present in the edge mapping image [23]. Kasim employed a hybrid spatial learning structure that integrates K-means clustering and expectation maximization to pinpoint the region of interest, thereby effectively mitigating the influence of staining and illumination on detection. Cheng introduced an innovative fuzzy morphological neuron model network that converts images from the RGB color space to the HSL color space, utilizing a fuzzy morphological network to identify white blood cells [24]. Lin et al. proposed a sophisticated white blood cell extraction algorithm based on feature weight adaptive K-means clustering. Prior to extracting white blood cells, they combined color space decomposition and K-means clustering for image segmentation, subsequently employing the watershed algorithm to separate complex white blood cells, ultimately achieving classification.

While these methods can effectively detect blood cells, they typically necessitate the completion of cell color space conversion or segmentation prior to detection. Furthermore, the precision of detection is often contingent upon the outcomes of image processing. These techniques also entail intricate operational procedures, which can render cell detection laborious and consequently diminish its efficiency. Consequently, the utility of these methods in clinical diagnosis is constrained.

#### 2.2.2. Methods Based on Deep Learning

Currently, the utilization of deep learning in target detection algorithms predominantly falls into two categories. The first category encompasses single-stage detection algorithms, exemplified by the YOLO series and SSD. The second category comprises two-stage detection algorithms, with Faster-R-CNN being a notable representative. This algorithm identifies potential target regions via the region proposal network. Liu et al. employed an enhanced Faster-R-CNN to detect and count red blood cells, demonstrating its efficacy in identifying such cells [25]. However, the detector’s demand for substantial computing resources and its relatively low detection rate are notable drawbacks. Moreover, it exhibits a higher missed detection rate when dealing with overlapping and densely populated areas. Zhang et al. proposed a density estimation method based on YOLO for cell counting, enhancing cell detection capabilities by modifying the backbone network of YOLO. Despite achieving certain improvements in the detection rate, the simplicity of the YOLO series’ network structure limits its feature extraction ability. Furthermore, the combination of neck convolution and upsampling fails to fully incorporate high-quality context information, thereby affecting the overall detection accuracy.

## 3. Method

To address the issues identified in current research, this paper introduces a blood cell detection algorithm utilizing SCD-YOLOv7. The primary enhancements are as follows:

In the initial stage, the C-MP (Cross-Modality-Projection) module and the D-ELAN (Dual-Evolving Layer-Aggregation-Network) module are used, which work together in the feature extraction network. They not only focus on the channel dimension but also take into account the characteristics of the spatial dimension, thus optimizing the process of feature extraction and enhancing the recognition ability of overlapping cell features.

Subsequently, the Sim-Head (Simplified Detection Head) detection module is presented. This module is designed for the optimal utilization of the global information embedded in the deep feature map and the local nuances in the shallow feature map. Such an approach markedly improves the algorithm’s efficacy in detecting small-sized targets.

In conclusion, the Focal-EIoU (Enhanced Intersection over Union) loss function is employed. This not only expedites the model’s convergence process but also markedly enhances the accuracy of blood cell detection.

Through quantitative and qualitative analysis of ablation experiments and comparative experimental results, the detection accuracy of the SCD-YOLOv7 algorithm on the blood cell data set reached 92.4%, increased by 7.2%, and the calculation amount was reduced by 14.6 G.

**Figure 1:**
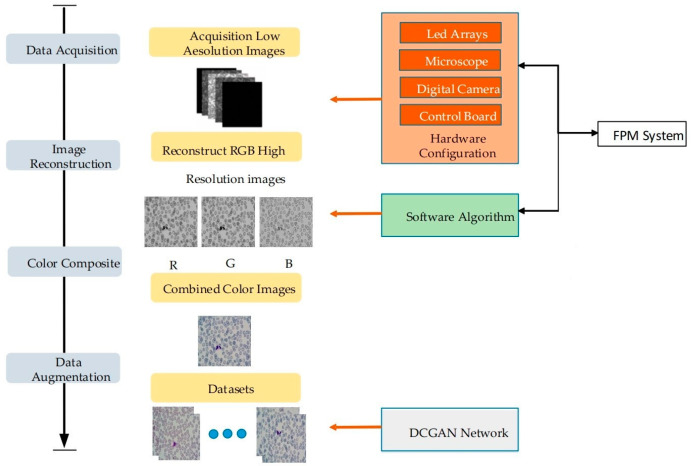
Data set production of Fourier compression microscopy imaging technology.

**Figure 2:**
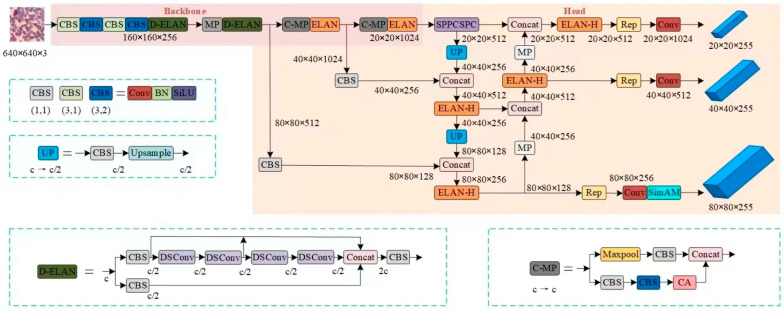
SCD-YOLOv7 network structure.

In this study, the issue of blood cell detection using Fourier ptychographic microscopy is delved into. Initially, low-resolution images from the three RGB channels of the blood cells were captured using the FPM system’s experimental platform. These were subsequently reconstructed to yield high-resolution and large-field images of the blood cells. Following this, preprocessing steps were applied to the gathered images. To tackle this challenge, an efficient neural network classifier has been developed based on the PyTorch framework. The specific process is depicted in Figure 1 and Figure 2.

To address this challenge, the data set was constructed using FPM technology, and the light source of the experimental platform was replaced with a programmable LED array illumination module. Utilizing MATLAB software, the LED lamps were precisely controlled to achieve multi-angle lighting. As for image acquisition, the DMK33UX264 camera was selected and successfully used to capture a substantial number of low-resolution images containing white blood cells for storage. Subsequently, the FPM algorithm was applied to reconstruct the RGB three-channel low-resolution images, resulting in high-resolution cell images. Necessary preprocessing was conducted on peripheral blood cell images to establish a basic data set. During the dataset creation process, blank samples and those lacking white blood cells were eliminated. Firstly, 200 original blood cell images were manually labeled, and then these labeled images were input into the network for training, and a prediction model was obtained. Then, this model is used to detect the remaining 4800 images and export the TXT file containing the prediction results. After verification, the final blood cell image data set was constructed, including 5000 images. The data set production process of this experiment was assisted by pathologists to ensure the accurate labeling of data samples. Subsequently, the enhanced data were divided into training and test sets according to a specific proportion.

The Fluorescence Photometry Method (FPM) was employed to gather data on blood cells. Through meticulous data processing, a comprehensive dataset was derived, and the blood cell dataset was subsequently constructed. Utilizing the experimental platform of the FPM system, low-resolution images of three RGB channels of blood cells were collected. These images were then reconstructed to yield high-resolution images with a broad field of view. Prior to training, four enhancement strategies were applied to the blood cell image data to enhance the model’s generalization and robustness. Following this, the refined YOLOv7 network was utilized for blood cell detection. The structure of the improved YOLOv7 network is depicted in Figure 2.

### 3.1. SCD-YOLOv7 Detection Model

**Figure 3:**
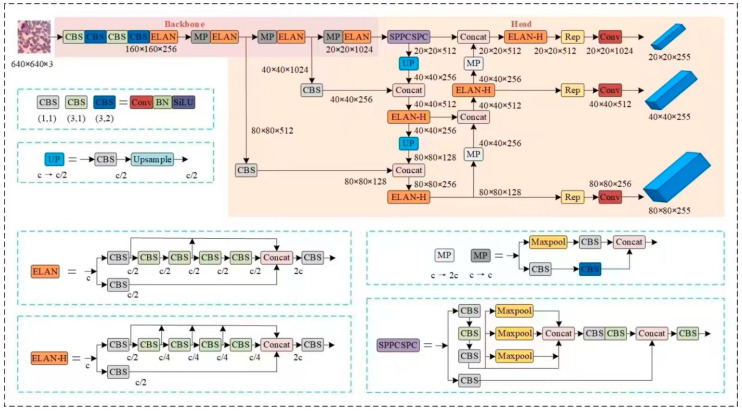
The network structure of YOLOv7.

YOLOv7 is a high-performance deep learning model for object detection. In tests conducted on the COCO public dataset, this model demonstrated superior detection accuracy and speed. The network structure of YOLOv7 comprises three components: Input, Backbone, and Head. Initially, the input section scales the image size to 640 × 640 × 3, which is then sent to the feature extraction network. This network includes several CBS modules, ELAN modules, and MP modules. In the prediction phase, feature maps of large, medium, and small scales are obtained through multi-scale feature fusion facilitated by the FPN (Feature Pyramid Network) and PAN (Path Aggregation Network) structures. These feature maps are subsequently sent to the detection head to generate three-scale prediction results. The final detection results are obtained through non-maximum suppression processing. The network structure of YOLOv7 is depicted in Figure 3.

**Figure 4:**
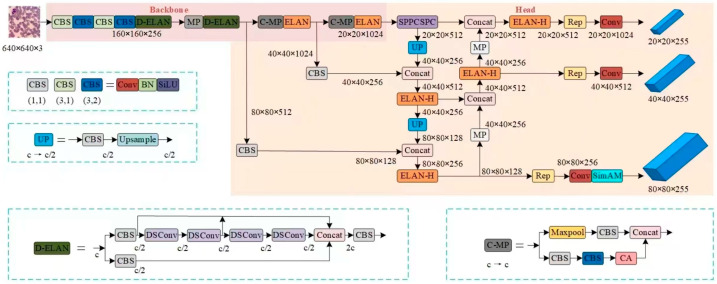
The network structure of SCD-YOLOV7.

This chapter addresses the challenges of detecting red blood cells, which often constitute a large proportion of small targets in blood cell images, as well as the complexities of image backgrounds and overlapping cells. To achieve precise detection of both red and white blood cells, we propose a blood cell image detection model based on an enhanced version of YOLOv7-SCD-YOLOv7. The network structure of the SCD-YOLOv7 model is depicted in Figure 4.

#### 3.1.1. Feature Extraction Network

Blood cell imaging, an acquired optical microscopic image, presents a complex background and overlapping cells. This complexity often results in a significant number of invalid background features extracted by the YOLOv7 model, thereby hindering blood cell detection. Concurrently, the use of intricate models with numerous parameters escalates the demand for computational resources and heightens the risk of overfitting. To address these issues, the integration of the C-MP module and D-ELAN module is proposed. These modules consider the characteristics of both channel and spatial dimensions, effectively broadening the scope of feature information. They optimize the feature extraction process and enhance the ability to extract features from overlapping cells.

**Figure 5:**
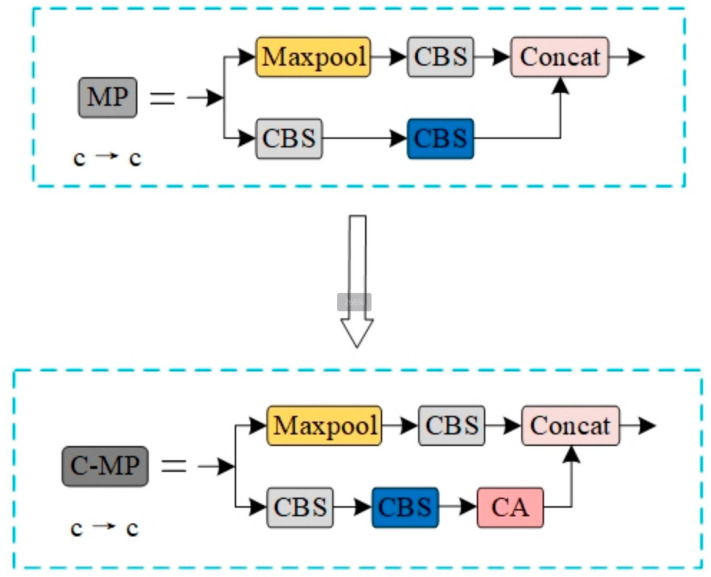
MP module before and after improvement.

Specifically, the coordinate attention mechanism (CA) is fused in the MP structure, and the output feature map is passed to the CA attention mechanism after 3 × 3 convolution. This fusion enables the network layer to simultaneously learn rich channel and spatial attention weights, effectively retaining complex feature information. It not only improves the model’s ability to locate overlapping cells but also can extract the key feature information of the image more accurately in the feature extraction stage. Since the CA module requires a certain amount of computational overhead, in order to save computational costs, this chapter only adds C-MP modules at key locations in the backbone network. This not only ensures the efficiency of the model on the blood cell data set but also improves the detection accuracy. The C-MP module structure is shown in Figure 5.

**Figure 6:**
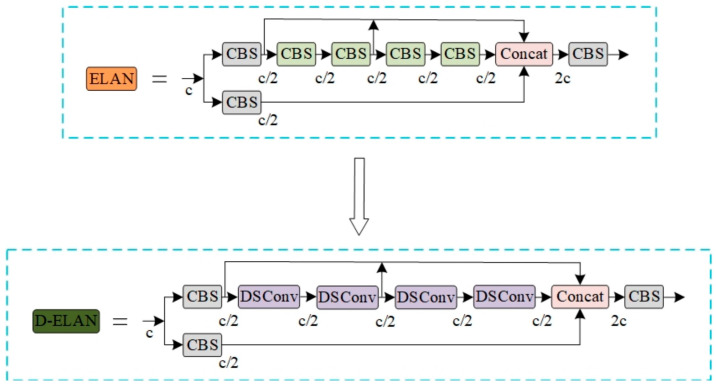
ELAN module before and after improvement.

When analyzing the model, it is noted that although the improved C-MP module effectively improves the accuracy and recall rate of the model, there are also problems with increasing the number of parameters and increasing the complexity of the model. In order to balance the accuracy and model complexity, distributed shift convolution (DSConv) is introduced to further optimize the model structure. Through the analysis of the standard convolution, the ELAN module is improved, and the D-ELAN structure is proposed, as shown in Figure 6. The D-ELAN structure improves the convolution kernel from the second layer to the fifth layer to DSConv so as to improve the model detection speed and reduce the number of parameters. This improvement makes the SCD-YOLOv7 model more efficient and lightweight while maintaining high performance and is suitable for complex tasks such as blood cell image detection.

#### 3.1.2. Sim-Head Detection Head

**Figure 7:**
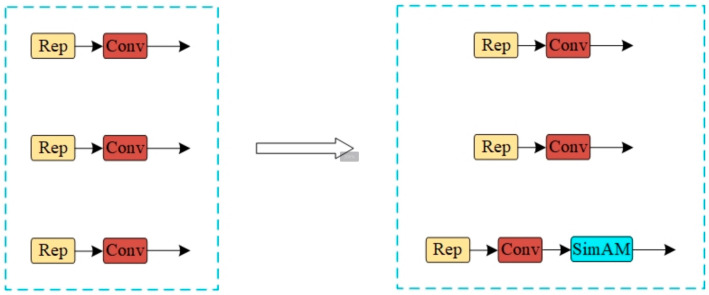
Sim-Head detection head.

Addressing the challenges of densely distributed small targets and the potential loss of detailed information in blood cell image detection, we introduce a non-parametric attention module and design a detection head, Sim-Head, specifically for small target identification. The structure is depicted in Figure 7. The Sim-Head detection head optimally utilizes the information produced by the feature fusion network, thoroughly investigates the relationship between pixel features, and exhibits heightened sensitivity to small targets, particularly in scenarios with densely distributed small targets. The enhanced Sim-Head detection head not only elevates the model’s detection accuracy but also bolsters its robustness, ensuring stable detection performance even under complex backgrounds and cell overlap conditions.

**Figure 8:**
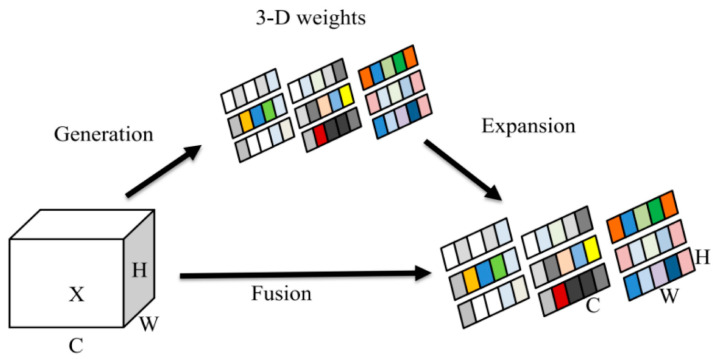
SIMAM attention mechanism.

The SIMAM module is a streamlined attention mechanism. Unlike existing channel and spatial attention mechanisms, it can learn attention across both channels and spaces, thereby capturing key information in the feature map with greater flexibility. As a non-parametric 3D attention module, SIMAM diverges from 1D and 2D attention mechanisms that solely focus on the importance of channels or spaces. It can concurrently emphasize the importance of each channel and spatial location feature, inferring the three-dimensional attention weight through an in-depth analysis of feature mapping. In visual tasks, this mechanism assigns weights to neurons based on their importance, thereby directing more attention to information-rich neurons and significantly enhancing the model’s feature extraction capability. The structure of the SIMAM attention mechanism is depicted in Figure 8. The module not only takes into account the correlation between space and channel dimensions but also does not increase the number of additional parameters, enabling efficient and accurate detection.

#### 3.1.3. Loss Function Optimization

The efficacy of the YOLOv7 algorithm is significantly influenced by the bounding box loss function. The CIoU loss function, when applied to address the imbalance between positive and negative samples, results in a decrease in detection accuracy. To mitigate this issue, a novel loss function is presented in this chapter, Focal-EIoU, which integrates EIoU Loss and Focal Loss. The EIoU Loss component more precisely calculates the overlap between bounding boxes, thereby providing more accurate metrics for the loss function. On the other hand, Focal Loss aims to counteract the imbalance between positive and negative samples by diminishing the weight of samples that are prone to misclassification. This reduces the detrimental impact of negative samples on model training. The combined Focal-EIoU loss function not only optimizes the calculation method of bounding box overlap but also alleviates the imbalance between positive and negative samples by adjusting the sample weight, thereby enhancing the model’s detection performance. The Focal-EIoU loss function is defined as follows:

(1) $$L_{EIOU}=1-IOU+\frac{\rho^{2}\left(h,b^{gt}\right)}{{\left(w^{c}\right)}^{2}+{\left(h^{c}\right)}^{2}}+\frac{\rho^{2}\left(w,w^{gt}\right)}{{\left(w^{c}\right)}^{2}}+\frac{\rho^{2}\left(h,h^{gt}\right)}{{\left(h^{c}\right)}^{2}}$$

In the formula, and denotes the width and height of the minimum circumscribed rectangle that can cover both the true box and the prediction box, and the function is used to calculate the Euclidean distance between the two points. The EIoU function significantly reduces the difference between the target bounding box and the real bounding box, thereby improving the positioning accuracy of the target detection.

The mathematical expression of the Focal Loss function is as follows: It makes the loss of positive samples occupy a larger proportion of the total loss through a specific weight adjustment mechanism and optimizes the training process of the model. It not only solves the problem of gradient explosion in small target detection but also improves the detection ability of the model for targets of different sizes.

In the formula, the constant is used to balance the numerical range of the loss function and ensure the stability of the training. By integrating EIoU Loss and Focal Loss, the final Focal-EIoU loss function is obtained.

In this chapter, the loss function is optimized to improve the accuracy of regression and the convergence speed of the model in the blood cell detection task. The improved loss function is Focal-EIoU, which inherits the advantages of the EIoU loss function in measuring the geometric difference of the bounding box. By explicitly considering the three key factors of overlapping area, center point, and side length, the model returns the position and size of the bounding box more accurately when locating the target. In addition, by integrating the idea of Focal Loss, the loss function further enhances the model’s attention to high-quality Anchors, so that the model can quickly learn effective feature representations during the optimization process, thereby improving the accuracy of target detection.

## 4. Experiment and Results

### 4.1. Experimental Environment and Configuration

In this experiment, Python 3.8 is used as the compiled version of the code, and Pytorch1.13.0 is used as the deep learning framework. Code debugging is performed in the Windows 10 environment. In order to shorten the model training time, training is performed on the cloud server. This chapter uses Intel (R) Xeon (R) CPU E5-2686 v4 processor, 3060 graphics card (12 GB memory), and 30 GB memory. All experiments are implemented using the PyTorch 1.13.0 and CUDA11.7.0 framework. The experimental environment is shown in Table 1.

In this experiment, the pre-training weight file used in the YOLOv7-main/train.py file is modified to be YOLOv7.pt, the image loading size is set to 640 × 640, and the data loading file is set to myYOLOv7.yaml. The SGD optimizer with simple, efficient, flexible, and good generalization ability is selected. In view of the limitation of computer resources and memory, the batch size is selected to be 2, and the initial learning rate, periodic learning rate, and weight attenuation coefficient are set as common default values. The specific hyperparameters are set as shown in Table 2.

### 4.2. Experimental Analysis

#### 4.2.1. Data Enhancement Strategy Results Analysis

In this section, four enhancement strategies of Hsv, Flip, Mixup, and Mosaic are used to increase the diversity of samples. In order to verify the effect of the data enhancement strategy on the detection performance of the SCD-YOLOv7 model in the blood cell image data set, experiments are carried out. The results are shown in Table 3.

#### 4.2.2. Analysis of Improved Detection Head Results

In this paper, the small target detection head based on the SimAM non-parametric attention mechanism is optimized and improved. In order to further verify the effectiveness of the Sim-Head detection head, this paper also compares it with the detection head structure that introduces attention mechanisms such as SE, CBAM, and ECA. The experimental results are shown in Table 4, and the mAP of the improved model has an increase of three or four percentage points in WBC/RBC/ALL, which proves the superiority of the proposed improvement strategy. The detection head Sim-Head based on the non-parametric attention mechanism of SimAM can better capture the local features of the image, fully combine the deep features and shallow features, and make use of the advantages of both, so that the model has better detection ability in the detection scene where small targets are densely distributed.

#### 4.2.3. Analysis of Optimization Loss Function Results

In this section, based on the improved detection head SimAM and feature extraction network, the IoU loss function is optimized, and the original CIoU is improved to Focal-EIoU. During the experiment, the performance of Focal-EIoU in blood cell target detection was not only tested but also compared with other loss functions. Through comparative experiments, it is found that CIoU performs well in accuracy, while the Focal-EIoU loss function performs better in improving the mAP value. The experimental results are shown in Table 5; the improved loss function has an increase of two to three percentage points compared with the original model. Choosing Focal-EIoU as the improved loss function not only helps to speed up the convergence of the model but also significantly improves the detection accuracy of the model.

The parameters in the original Focal loss are used to adjust the weight distribution of positive and negative samples in the total loss. In the target detection task, the number of negative samples often far exceeds the number of positive samples, which may lead to the problem of sample imbalance during training. By selecting the value of the parameters, the influence of positive and negative samples on loss can be balanced, and the training effect of the model can be optimized. At the same time, parameters play an important role in adjusting the weight of difficult and easy samples in Focal loss. During the training process, the influence of samples with different difficulty levels on model optimization is also different. By adjusting the parameters, the model can focus on those samples that are difficult to distinguish, thereby improving the robustness and generalization ability of the model. In order to determine the optimal combination of parameters, multiple sets of experiments are carried out, and the experimental results are shown in Table 6.

For different combinations of values, the average accuracy of the SCD-YOLOv7 model for white blood cell and red blood cell detection is different. Due to the infinite value of the combined value, this paper does not carry out large-scale value experiments. Only a few sets of values are selected within the appropriate range for experiments. Finally, the value combination with the parameter of 1 is selected.

#### 4.2.4. Analysis of Ablation Experimental Results

This section performs ablation experiments on the proposed improved SCD-YOLOv7 model on the data set to verify the impact of each improved module on the network model. The ablation experiment is carried out based on data enhancement. The detection head Sim-Head with SimAM attention mechanism, the C-MP module with CA attention mechanism, the D-ELAN module with improved standard convolution, and the optimization strategy of improved Focal-EIoU loss function are analyzed in detail. The experimental results are shown in Table 7.

Each independent improvement strategy shows good performance in terms of parameter quantity and calculation amount, but it is not the optimal choice. By integrating the improvements of the above four parts, the performance of the model is maximized. Compared with the original model, the improved model has a significant improvement in detection accuracy. Specifically, the improved Sim-Head detection head has the most significant improvement in the performance of the blood cell detection model, and the mAP value has increased by 1.9%. Secondly, the C-MP module in the feature extraction network is improved, and the mAP value is increased by 1.5%. In addition, the improved D-ELAN module not only improves the detection accuracy mAP by 0.7% but also greatly reduces the amount of calculation by 15.1G, which proves the effectiveness of the module in improving the performance of the model. The optimization strategy of the Focal-EIoU loss function is introduced, which makes the mAP value increase by 0.8% on the previous basis. Through the comprehensive application of these improvement strategies, the final average accuracy mean mAP value reaches 92.4%, which is 7.2% higher than that of YOLOv7, and the calculation amount is reduced by 14.6G. The experimental results show that the proposed method significantly improves the detection performance of blood cell images for the network model.

In the fifth line, the Focal IoU loss function is used with two parameters ranging from 36.5M to 36.4M, reducing the number of parameters. In the second to last line, the Focal IoU loss function is used with 36.5M parameters and 103.7G of computation. In the last line, the Focal IoU loss function is used with 36.5M parameters and 88.6G of computation, without any change in the number of parameters. This is because in this case, the number of parameters remains unchanged, indicating that the network structure has not been further optimized or adjusted when introducing the Focal IoU loss function. This is because a balance point has been reached, which is to improve model performance by optimizing the loss function without changing the number of parameters.

#### 4.2.5. Analysis of Comparative Experimental Results

In order to evaluate the detection effect and performance of the model more comprehensively, the experimental results of the improved model SCD-YOLOv7 were quantitatively and qualitatively analyzed. In addition, with other commonly used target detection models, under the same configuration conditions, the same data set is used for training and verification, and the detection results are compared comprehensively.

**Figure 9:**
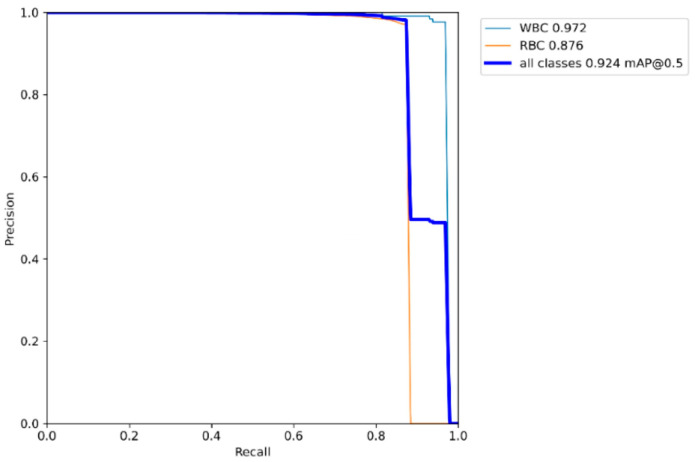
P-R curve of SCD-YOLOv7 model.

Quantitative results analysis: In order to objectively evaluate the detection performance of the SCDYOLOv7 model on the blood cell image data set, the P-R curve of the model is drawn, as shown in Figure 9. In the P-R curve, the abscissa represents the recall rate, the ordinate represents the accuracy rate, and the mAP@0.5 value of the cell target to be tested is the area enclosed by the curve and the coordinate axis. When the recall rate is close to 1, that is, the model can almost identify all blood cell targets, and the accuracy rate begins to decline rapidly. It fully proves the excellent performance of the SCD-YOLOv7 network in blood cell detection.

**Figure 10:**
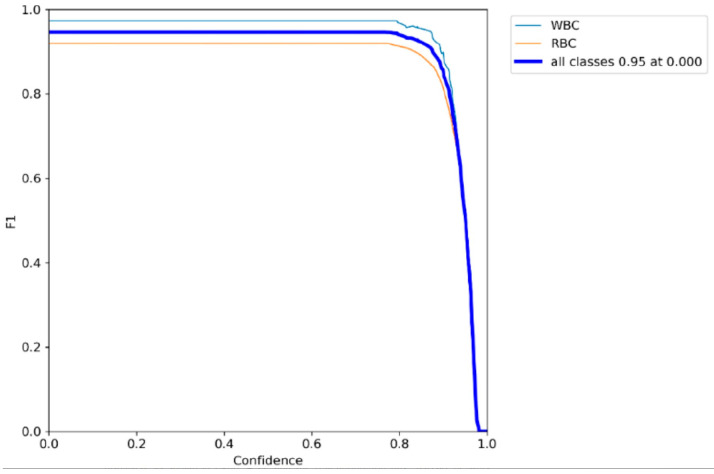
F1-scores curve of SCD-YOLOv7 model.

F1-scores are a comprehensive parameter to evaluate detector Recall and Precision performance. As shown in Figure 10, it is the F1 score change curve of the algorithm in this paper. The three curves in the figure represent the average F1 score change of white blood cell and red blood cell categories in the data set, respectively. The ordinate is the F1-scores value of each target, and the abscissa is the threshold range.

**Figure 11:**
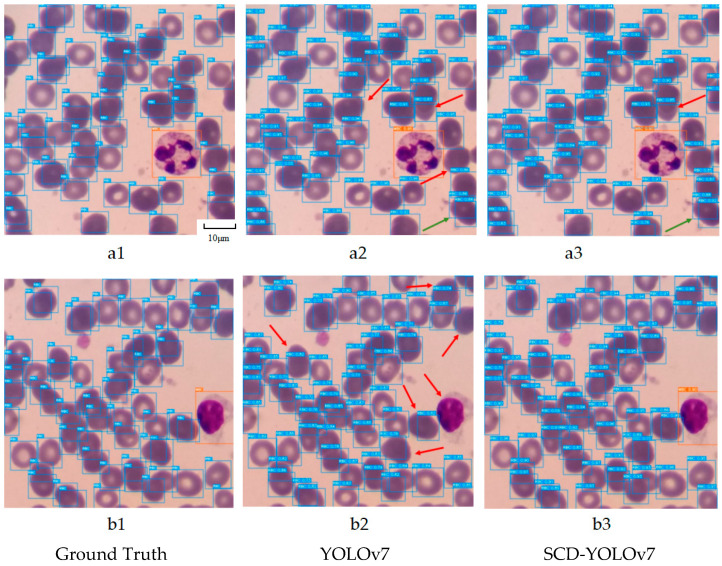
Blood cell detection effect diagram.

Qualitative result analysis: In order to intuitively show the actual performance of the SCD-YOLOv7 model proposed in this paper in the blood cell image detection task, two sets of images in the test set were tested and verified. The results are visually displayed, as shown in Figure 11, and the real annotation box, YOLOv7 network prediction results, and SCD-YOLOv7 network prediction results are compared.

In Figure 11(a1–a3), the YOLOv7 model has three cases of missed detection of red blood cell targets and one case of false detection. It is caused by the overlapping of red blood cell targets and the complex background in the blood cell image. In contrast, the improved SCD-YOLOv7 model has significantly improved the detection effect, with only one missed detection and one false detection, which reflects the robustness of the model in complex backgrounds. In addition, the confidence in the prediction results of the SCD-YOLOv7 model is generally higher than that of the YOLOv7 model, which verifies the effectiveness of the model in improving detection accuracy. In Figure 11(b1–b3), the prediction results of the YOLOv7 model are also unsatisfactory, and there are more red blood cells and white blood cells missed. However, the SCD-YOLOv7 model can completely predict all the cell targets to be detected, fully demonstrating the superior performance of the model in the blood cell detection task. This is due to the improvement of the small target detection head and the feature extraction network part, which makes the model more advantageous in capturing local information and global information.

In order to further verify the advantages of the SCD-YOLOv7 model proposed in this paper in the blood cell image detection task, in this paper, a comparison is made between the SCD-YOLOv7 model and other existing mainstream target detection models. The parameters, computational load, mAP values, and other performance indicators of these models are calculated and analyzed when they are applied to a blood cell dataset. The comparative experiments are carried out under the same experimental conditions and parameter configuration, and the results are shown in Table 8 the improved algorithm mAP/WBC increased from 75.8% to 97.2%, mAP/RBC increased from 71.8% to 87.6%, and mAP/ALL increased from 73.8% to 92.4% the detection performance is greatly improved compared with the original model.

The SCD-YOLOv7 improved algorithm proposed in this paper performs best on mAP, and the parameter quantity and calculation amount are excellent. For the YOLOv5 s model, although the parameter quantity and calculation amount are the lowest, which are 7.1 M and 16.3 G, respectively, its detection accuracy is only 82.3%. Compared with YOLOX, the SCD-YOLOv7 model has obvious advantages in detection accuracy, parameter quantity, and calculation amount. The mAP value is 5.9% higher than that of the YOLOX model, the parameter amount is reduced by 17.7 M, and the calculation amount is reduced by 67 G. Compared with the YOLOv6 model, the accuracy mAP value is increased by 7.7%. Compared with other algorithms, YOLOv5l, YOLOv4, Faster-RCNN, and SSD, the proposed improved model SCD-YOLOv7 has the lowest computational complexity, and the mAP values are increased by 6.6%, 10.8%, 13.9%, and 18.6%, respectively. Compared with all the above algorithms, the model proposed in this paper performs well in parameter quantity and calculation amount for blood cell detection, and the detection accuracy reaches 92.4%. At the same time, the effectiveness and feasibility of the improved algorithm in blood cell image detection are verified.

## 5. Summary

In this paper, a blood cell detection algorithm based on SCD-YOLOv7 is proposed to solve the problems of small red blood cell targets, complex image backgrounds, and cell overlap in the blood cell image cell detection task. Firstly, the framework of YOLOv7 and the improved blood cell image detection model SCDYOLOv7 are introduced, which are described from four aspects: feature extraction network, small target detection head, and loss function. Then, the parameter setting, experimental environment, and performance evaluation index are defined. Different improvement strategies are tested, and the ablation experimental results of each module are analyzed in detail. Finally, the experimental results on the blood cell image data set are quantitatively and qualitatively analyzed. Under the same configuration conditions, the detection effect is compared with other commonly used detection models, which proves the effectiveness of the model proposed in this paper.

## Figures and Tables

**Table 1:** Experimental environment requirements.

Name	Parameter Description
Operating system	Windows10
CPU	Intel(R) Xeon(R) CPU E5-2686 v4
GPU	3060-12G
Memory	30G
CUDA Version	11.7
Python	3.8
Pytorch	1.13.0

**Table 2:** Super parameter settings.

Name	Numerical Value
Image size	640 × 640 × 3
Batch size	2
lr0	0.01
lrf	0.1
Optimizer	SGD
Weight decay	0.0005
Momentum	0.937

**Table 3:** Experimental results of data augmentation strategy.

Network Model	Parameter Quantity (MB)	Computation (GB)	mAP (WBC)	mAP (RBC)	mAP (ALL)
Yolov7	36.5	103.2	85.1	85.3	85.2
Yolov7 + Flip	36.5	103.2	85.6	85.2	85.4
Yolov7 + Flip + Mixup	36.5	103.2	86.1	85.4	85.7
Yolov7 + Flip + Mixup + Hsv	36.5	103.2	86.4	85.7	86.0
Y7 + Flip + Mixup + Hsv + Mosaic	36.5	103.2	87.3	85.6	86.5

**Table 4:** Comparison of different attention mechanism experiments.

Attention Mechanism	Parameter Quantity (MB)	Computation (GB)	mAP (WBC)	mAP (RBC)	mAP (ALL)
Yolov7	36.5	103.2	87.3	85.6	86.5
Yolov7 + SE	36.5	103.7	87.4	86.9	87.2
Yolov7 + CBAM	36.5	103.7	92.6	83.7	88.2
Yolov7 + GAM	38.2	103.7	89.7	86.0	87.8
Yolov7 + ECA	36.5	103.6	88.9	83.9	86.4
Yolov7 + BiFormer	36.8	103.7	85.8	81.8	83.8
Yolov7 + SimAM	36.5	103.6	90.4	86.4	88.4

**Table 5:** Loss function experimental comparison.

Loss Function	Parameter Quantity (MB)	Computation (GB)	mAP (WBC)	mAP (RBC)	mAP (ALL)
GIoU	36.5	88.6	93.3	87.0	90.2
DIoU	36.5	88.6	94.2	86.9	90.5
CIoU	36.5	88.6	95.7	87.4	91.6
WIoU	36.5	88.6	93.2	87.5	90.4
EIoU	36.5	88.6	95.9	86.9	91.4
Focal-EIoU	36.5	88.6	96.9	87.4	92.2

**Table 6:** Experimental results of different Focal-EIoU parameters.

a	g	Parameter Quantity (MB)	Computation (GB)	mAP (WBC)	mAP (RBC)	mAP (ALL)
1	1	36.5	88.6	97.2	87.6	92.4
1	0.75	36.5	88.6	96.0	86.4	91.2
1	0.5	36.5	88.6	96.8	87.4	92.1
2	1	36.5	88.6	94.2	87.0	90.6
2	0.75	36.5	88.6	95.8	87.2	91.5
2	0.5	36.5	88.6	95.4	86.8	91.1

**Table 7:** Ablation experiment.

Y7 + Data	Sim-Head	C-MP	D-ELAN	Focal-EIoU	Parameter (MB)	Computation (GB)	mAP (WBC)	mAP (RBC)	Map (ALL)
Y					36.5	103.2	87.3	85.6	86.5
Y	Y				36.5	103.6	90.4	86.4	88.4
Y		Y			36.5	103.2	89.0	86.8	87.9
Y			Y		36.4	88.2	85.9	85.0	85.5
Y				Y	36.4	103.2	90.5	84.3	87.4
Y	Y	Y			36.5	103.7	93.5	88.2	90.9
Y	Y	Y	Y		36.5	88.6	95.7	87.4	91.6
Y	Y	Y	Y	Y	36.5	88.6	97.2	87.6	92.4

**Table 8:** Experimental comparison of different network models.

Network Model	Parameter Quantity (MB)	Computation (GB)	mAP (WBC)	mAP (RBC)	mAP (ALL)
SSD	24.0	274.5	75.8	71.8	73.8
Faster-RCNN	136.7	401.8	81.3	75.7	78.5
YOLOv4	52.4	119.7	85.1	78.0	81.6
YOLOv5l	46.3	108.9	86.7	85.0	85.8
YOLOv5s	7.1	16.3	87.4	77.3	82.3
YOLOX	54.2	155.6	87.3	85.6	86.5
YOLOv6	34.8	85.6	86.8	82.7	84.7
YOLOv7	36.5	103.2	85.1	85.3	85.2
SCD-YOLOv7	36.5	88.6	97.2	87.6	92.4

## Data Availability

Data are contained within the article.
